# ‘I Will Never Succeed’: An Inductive Thematic Analysis of Psychosocial Challenges Experienced Before Sleeve Gastrectomy Among Patients in Turkey

**DOI:** 10.1177/23743735261458021

**Published:** 2026-07-03

**Authors:** Sabahat Ölcer, Mehmet Tayanc

**Affiliations:** 1 Department of Palliative Medicine, University Medical Center Göttingen, 9375 University of Göttingen, Göttingen, Germany; 2 Department of Human-Centered Technology Development, Institute of Computer Science, Campus Bottrop, Ruhr West University of Applied Science, Bottrop, Germany; 3 Department of Sociology, Faculty of Arts and Sciences, 187476 Siirt University, Siirt, Turkey

**Keywords:** obesity, internalised stigma, self-perception, emotional eating, sleeve gastrectomy, psychosocial factors

## Abstract

Our study explored psychosocial experiences in the pre-surgery period that shaped the decision to undergo sleeve gastrectomy, with a focus on self-perception and internalised stigma. Nineteen participants from Turkey were purposively recruited. Semi-structured in-depth interviews were conducted to collect data, which were then analysed thematically with an inductive approach. Seven key themes emerged: body image, social stigma, family dynamics, emotional coping, psychological barriers to weight loss, surgical decision-making, and psychosocial impacts of weight. Participants reported feelings of shame, frustration, and social withdrawal linked to self-blame. Emotional eating was a common coping mechanism, while repeated dieting led to demotivation and exhaustion. Fear and hope influenced surgery decisions, with information seeking alleviating uncertainty. Family relationships both supported and stressed participants, affecting their emotions and health behaviours. These findings underline the complex psychosocial challenges faced before surgery and their influence on decision-making, emphasising the need for supportive and stigma-free care.

## Introduction

Obesity is a disease that has serious effects on a person’s physical, psychological, and social health.^1^ Weight gain and its management affect how people perceive themselves, handle their feelings, and interact with others.^2,3^ Sleeve gastrectomy is now one of the most commonly performed surgical treatments for morbid obesity.^4^ When people consider sleeve gastrectomy, they often face weight stigma, family issues, and psychosocial problems, which shape both how they relate to food and how they see themselves.^2,5-7^

Studies have revealed that weight stigma both contributes to obesity and results from it, leading to ongoing cycles that affect psychosocial health and treatment outcomes.^8,9^ Negative attitudes towards people with obesity are observed in healthcare settings, workplaces, and interpersonal relationships.^2,8^ These experiences can lead to emotional distress, low self-esteem, and maladaptive coping behaviours, such as emotional eating.^2,10^ Stigma increases the emotional burden of obesity and perpetuates it by reinforcing unhealthy eating habits and reducing engagement in health-promoting behaviours.^3^

Emotional eating is a common behavioural pattern among people with obesity and occurs in response to negative emotions such as stress, anxiety, or sadness.^11^ Difficulties in emotion regulation and maladaptive coping behaviours are key factors in the development and maintenance of emotional eating, which can lead to repeated cycles of weight gain and emotional distress.^11,12^ These patterns can become more complex when disordered eating behaviours, such as binge eating disorder, are present. This condition frequently occurs alongside obesity and is linked to higher psychosocial health risks.^13-15^

Although weight loss is often viewed as a matter of self-control, psychological barriers, emotional difficulties, and social pressures play a major role in this process.^16,17^ Many people experience repeated unsuccessful dieting attempts, which can cause hopelessness and frustration.^18^ In this context, sleeve gastrectomy may offer a new option and source of hope for people with obesity.^19^ Reasons for choosing surgery include both physical and psychosocial factors, such as improving mobility, reducing health problems, and enhancing quality of life.^16^ However, these reasons may coexist with emotional ambivalence, concerns about surgical risks, and uncertainty about postoperative outcomes.^17,20^

Psychological and social support before and after sleeve gastrectomy is important for optimal outcomes. Studies have highlighted the need to address psychological concerns, difficulties in emotion regulation, and concerns about body image.^3,21^ Emotional health influences adherence to lifestyle changes and is linked to long-term weight loss and better quality of life.^22,23^ Family and social relationships can either facilitate or hinder psychological adjustment more difficult, depending on the support, empathy, and understanding they provide.^23,24^ For example, families may unintentionally reinforce negative emotions or unhealthy eating habits through well-intentioned but emotionally invalidating comments.^6^

Although awareness of the psychological and social challenges around bariatric surgery is growing,^5,21,24^ the pre-surgery psychosocial processes leading to the decision remain underexplored. This gap highlights the need to examine how psychosocial experiences during the pre-surgery period influence individuals’ decisions to undergo sleeve gastrectomy. Understanding these psychosocial challenges is crucial for informing tailored pre-surgical support and improving patient outcomes. Our study focuses only on the pre-surgery period and specifically aims to explore the psychosocial factors influencing the decision process to undergo sleeve gastrectomy. By retrospectively examining the motivations, emotional challenges, and social contexts prior to surgery, our research intends to better understand individuals’ experiences and needs at this important stage. The findings aim to guide more supportive and effective pre-surgical care and promote psychological health.

## Methods

### Research Team

Both authors hold PhD degrees and have expertise in qualitative research methods. One author specialises in emotions, immigration, and digital health and actively conducts research in these areas. The other author focuses primarily on ethnographic research and has extensive experience in this area. Both researchers engaged in reflexive practice, considering how their professional backgrounds, prior experiences, and personal perspectives might influence the interview process, participants’ responses, and interpretation of the data.

### Study Design

This exploratory qualitative study aimed to understand participants’ psychosocial experiences that shaped their decisions to undergo sleeve gastrectomy. We used an inductive thematic analysis to explore the experiences of participants without imposing pre-existing theoretical frameworks, allowing themes to emerge from their narratives.^25,26^ This approach enabled us to gain deeper insight into their experiences and emotional responses. Data collection involved semi-structured in-depth interviews.

The interview guide included questions about basic demographic information and data required to calculate Body Mass Index (BMI) before sleeve gastrectomy. It explored participants’ emotional, psychological, and social experiences that shaped their perspectives and informed their decision-making during the pre-surgery period. The guide included broad open-ended questions to encourage detailed narratives, supported by specific prompts addressing key areas such as history of obesity, personal meanings attributed to weight gain, perceptions and feelings related to weight gain, emotional responses and psychological difficulties experienced over time, coping strategies, daily lifestyle (including diet and physical activity), social relationships, expectations regarding surgery, motivating and limiting factors in decision-making, and pre-surgical challenges. These open-ended questions were designed to explicitly capture participants’ psychological experiences prior to sleeve gastrectomy and to clarify how these experiences influenced their consideration of surgery.

On average, each interview lasted 41 minutes. Ethical approval for our study was granted by the Institutional Review Board on December 13, 2023 (Approval No. 6027). Our study was conducted in accordance with the Consolidated Criteria for Reporting Qualitative Research (COREQ) checklist. To protect participants’ anonymity and confidentiality, each participant was assigned a code (SG1 – SG19; SG: Sleeve Gastrectomy), which is used throughout the study.

### Recruitment

Most participants were recruited from different provinces in Turkey using a snowball sampling technique. Because this population was difficult to access, we also collaborated with community contacts (locators) who helped identify and approach potential participants. In addition, some participants were connected through social media platforms, where they sought information about the surgery and shared their experiences, further facilitating recruitment.

### Data Collection

We conducted semi-structured in-depth interviews that allowed in-depth exploration of participants’ personal experiences and perspectives. Data were collected from May 2024 to November 2024 through both face-to-face and online interviews, depending on participants’ location and availability. Interviews continued until data saturation was reached, defined as the point at which no new themes emerged in three consecutive interviews. After the 19th interview, no new themes were identified, and the data started to repeat, indicating that saturation had been achieved.

### Data Analysis

An inductive thematic analysis, as proposed by Braun and Clarke,^25^ was used to analyse the data. The analysis was supported by MAXQDA 24.9 software package. SÖ and MT conducted all interviews in Turkish. MT transcribed the interviews verbatim, and SÖ reviewed all transcripts for accuracy. The analysis was carried out in Turkish by SÖ to preserve participants’ original meanings. Only selected excerpts were translated into English by the research team, who are native Turkish speakers with extensive experience in qualitative research. This ensured both linguistic and cultural accuracy in translation. The translated excerpts were cross-checked against the original transcripts to preserve the nuance and authenticity of participants’ voices.

The analysis began with multiple readings of the transcripts to become familiar with the data. Codes were created inductively from the data, without reference to pre-existing theories, and were then grouped into possible themes based on conceptual similarities. As coding progressed, each interview was compared with previous ones to identify new codes or themes. This iterative process supported the assessment of data saturation. The initial results were discussed among the authors, and any disagreements were resolved by consensus. The final themes were selected based on their frequency and relevance to the study objectives.

### Rigor and Trustworthiness

The analysis followed Braun and Clarke’s^25^ recommendations for conducting reflective thematic analysis to ensure rigor and trustworthiness. Transparency was maintained by keeping detailed records of all analytic steps, from initial coding to theme development. Researcher reflexivity was emphasised throughout the study to acknowledge and minimise potential biases in data interpretation. During data collection and analysis, reflexive discussions helped ensure that interview questions, coding, and theme development remained aligned with psychosocial experiences relevant to decision-making for sleeve gastrectomy. Credibility was strengthened through ongoing discussion between the researchers, who collaboratively reviewed and refined themes. Representative participant quotes were used to support each theme and to ensure that findings remained closely grounded in participants’ lived experiences.

## Results

### Characteristics of the Participants

The participants were people living in Turkey who had sleeve gastrectomy surgery. In total, 19 people took part in the study (Table 1). Their ages ranged from 24 to 56 years (M = 37.9), and their average BMI before surgery was 44.4 kg/m^2^. The sample included 11 women and 8 men, and the majority were married (n = 14). Participants had diverse educational backgrounds. Eight participants held a bachelor’s or master’s degree, while the remaining participants had primary, secondary, high school, or vocational education. Five of the female participants were housewives, while others were employed in various occupations, including civil service, teaching, cosmetics, self-employment, and other professions. The participants had undergone sleeve gastrectomy between 1 and 5 years prior to the interviews, with an average time since surgery of 3.2 years.

**Table 1.** Participants’ Characteristics

Participant	Age	Gender	Educational attainment	Marital status	Occupation	Body mass index (BMI) before surgery
SG1	39	Female	Secondary school	Married	Housewife	40.1
SG2	35	Female	Bachelor degree	Married	Civil servant	42.1
SG3	28	Female	High school	Divorced	Civil servant	41.9
SG4	40	Female	Literate	Married	Housewife	37.5
SG5	27	Female	High school	Single	Student	45.4
SG6	41	Female	Bachelor degree	Married	Housewife	42.5
SG7	35	Male	Bachelor degree	Married	Self-employment	42.4
SG8	24	Male	Bachelor degree	Single	Civil servant	51
SG9	37	Male	Secondary school	Married	Worker	48.3
SG10	42	Female	Literate	Married	Housewife	38.6
SG11	25	Female	Bachelor degree	Single	Student	37.8
SG12	31	Female	Vocational school	Married	Cosmetician	41.7
SG13	28	Female	Bachelor degree	Single	Teacher	43.7
SG14	29	Female	Bachelor degree	Married	Housewife	40
SG15	46	Male	Master degree	Married	Civil servant	48.2
SG16	51	Male	Secondary school	Married	Photographer	40
SG17	55	Male	Secondary school	Married	Shopkeeper	51.4
SG18	56	Male	Primary school	Married	Driver	46.1
SG19	51	Male	Secondary school	Married	Retired	64.6

### Specific Themes

As a result of the analysis, we identified seven main themes reflecting participants’ complex psychosocial experiences related to their weight and associated challenges. These themes illustrate how participants interpreted their situation and how this process gradually influenced their consideration of sleeve gastrectomy prior to surgery. The themes are presented in Table 2 and supported by direct participants excerpts (see Supplementary File Table S1).

**Table 2.** Summary of Emergent Themes With Core Emotions, and Illustrative Quotations

Themes	Key emotions	Illustrative quotations (shortened)
Self-perception and emotional responses to weight gain	shame, frustration, helplessness, sadness, despair	“Up to a certain point, I did not really care about gaining weight, but one day I looked in the mirror and could not recognise myself. That day, I truly realised how much I had changed, and I felt very sad.” (SG8, male, 24, bachelor degree, civil servant)
Social experiences and stigma related to obesity	embarrassment, isolation, anger, humiliation	“It made me angry when people looked at me with pity. There were times when I was the subject of jokes. Even being called ‘fat’ had started to feel unbearable.” (SG19, male, 51, secondary school, retired)
Family dynamics and support	frustration, loneliness, validation	“My mom always said, ‘You are beautiful as you are,’ but I did not feel that way. Instead of offering support, they sometimes avoided talking about the issue altogether.” (SG18, male, 56, primary school, driver)
Emotional coping mechanisms and eating behaviours	relief, sadness, helplessness	“When I was upset, I would always turn to food. Even if I was full, my hands kept reaching for snacks. I ate to suppress my emotions.” (SG2, female, 35, bachelor degree, civil servant)
Psychological barriers to weight loss	hopelessness, exhaustion, ambivalence	“I dieted for 3 months and lost 20 kilos, but then I gained it all back. The constant hunger from dieting exhausted me.” (SG14, female, 51, bachelor degree, housewife)
Decision-making process and information seeking about surgery	fear, hope, anxiety, curiosity	“I always thought surgery was an unlikely option, but I wanted to lose weight and regain my mobility. When I heard an acquaintance had undergone surgery, I started considering it myself.” (SG13, female, 28, bachelor degree, teacher)
Impact of weight on psychosocial health	sadness, loneliness, inadequacy, despair	“I used to love going out, but as I gained weight, I started preferring to stay home. I isolated myself from people.” (SG15, male, 46, master degree, civil servant)

## Theme 1: Self-Perception and Emotional Responses to Weight Gain

Participants described gaining weight as a strongly emotional experience that changed how they saw themselves. Many felt shame and criticised themselves, blaming a lack of control and feeling frustrated by what they perceived as personal failure (SG1). This inner conflict led to a sense of helplessness, as participants felt unable to reverse the changes they had undergone. Over time, these struggles prompted participants to question the limits of their own efforts.

Many participants also described feeling increasingly disconnected from their own bodies, which intensified their emotional distress. Seeing themselves in the mirror often brought sadness and discomfort because they no longer recognised the person they saw, leading some to avoid mirrors and photographs altogether (SG2, SG8). These experiences were frequently marked by denial, emotional withdrawal, and despair. As this sense of disconnection persisted, some participants began to perceive sleeve gastrectomy as a more structured option for change.

## Theme 2: Social Experiences and Stigma Related to Obesity

The stigma associated with obesity had a strong impact on the social experiences of participants. Everyday situations, such as shopping or attending social events, frequently caused feelings of embarrassment and humiliation (SG1). Many participants became more aware of being judged or excluded because of their body size, which over time led to social withdrawal (SG12). Some felt anger and resentment when they became the target of jokes, pity, or undesirable comments (SG19). Although stigma was not always cited as a direct reason for seeking surgery, repeated experiences of social judgment and emotional strain led many to view sleeve gastrectomy as a potential way for relief and readiness for change.

## Theme 3: Family Dynamics and Support

Family interactions regarding weight gain evoked a mix of emotions, including both support and challenges. Many participants felt criticised or unsupported when family members gave advice about weight in a way that was not encouraging or was too critical (SG13). This criticism often led to frustration, as the advice was not always perceived as motivating and instead increased feelings of inadequacy (SG16).

Some participants experienced moments of validation and support, especially from family members who showed unconditional acceptance. However, when families avoided discussing weight openly or ignored the issue, participants felt isolated in their struggles (SG18). Navigating these dynamics, participants described emotionally demanding relationships that contributed to their broader re-evaluation of weight management options, including sleeve gastrectomy. These family challenges added to participants’ emotional strain.

## Theme 4: Emotional Coping Mechanisms and Eating Behaviours

Participants coped with emotional stress by turning to food, which created a complex link between their feelings and eating habits. Many relied on comfort foods to suppress negative feelings such as sadness, anxiety, or frustration (SG2, SG13, SG19), which brought short-term relief during distress (SG13, SG19). However, this temporary relief made controlling eating behaviour increasingly difficult, despite participants’ awareness of its adverse effects on health and well-being (SG2). As these cycles of distress and helplessness persisted, participants began relating surgery to their ongoing struggles with self-management and control. This further exacerbated the emotional exhaustion stemming from self-perception issues and repeated weight loss failures.

## Theme 5: Psychological Barriers to Weight Loss

Participants described psychological barriers that made weight loss feel unattainable. Experiences of unsuccessful attempts caused them to question the potential of their efforts and subtly shaped their openness to alternative solutions. Many described hopelessness after repeated dieting followed by weight regain (SG8, SG12). This cycle left them physically exhausted from constant dieting and emotionally drained by a goal that seemed impossible (SG14).

Many participants felt torn between their desire to lose weight and growing discouragement from repeated failures (SG19). This inner struggle made them hesitant to commit to long-term plans because they were afraid of being disappointed again. Some gave up altogether, believing that permanent weight loss was beyond their control, regardless of their efforts (SG8, SG12). These experiences helped participants realise their own limits and led them to see surgery as a more practical way to achieve their goals.

## Theme 6: Decision-Making Process and Information Seeking About Surgery

The decision to have weight loss surgery was often shaped by a complex mix of emotions. Fear and anxiety were common, as participants worried about the risks and challenges of the procedure (SG14, SG16). These feelings influenced how carefully they considered their options. Many felt uncertain about whether surgery was the right choice, which caused hesitation (SG13). Despite these fears, there was also strong hope; the chance for lasting weight loss and a better quality of life provided relief and motivation (SG16, SG19), which strengthened their desire to move forward despite ongoing doubts.

Curiosity played an important role in the decision-making process, as participants searched for information and spoke with people who had already undergone the surgery (SG7, SG13). Gaining knowledge reduced worries by clarifying expectations and framing surgery within the context of prior emotional and social struggles. Participants viewed this step not as a sudden decision but as the culmination of accumulated experiences over time.

## Theme 7: Impact of Weight on Psychosocial Health

For many participants, the emotional burden of weight changes manifested as profound sadness and loneliness, highlighting the strong link between weight and psychosocial health (SG3, SG15). This burden influenced how they evaluated weight management options over time. As their weight increased, participants experienced more frustration and a loss of identity (SG19). Many described no longer recognising themselves, both physically and emotionally, which led to a persistent sense of despair and helplessness. This detachment from self-image influenced participants’ openness to consider surgical intervention (SG3, SG19).

Feelings of inadequacy were also common, as participants blamed themselves for weight gain and struggled to meet societal expectations of health and beauty. These feelings increased their isolation and made it difficult to enjoy activities they once enjoyed (SG3, SG15, SG19). For several participants, the cumulative effect of emotional, social, and familial challenges helped them view surgery as a practical step toward regaining control and enhancing well-being.

## Discussion

Our study shows that the psychosocial effects of weight gain and obesity are significant during the pre-surgery period. Self-perception, internalised stigma, and social experiences play a central role in how participants perceive and respond to their health. Curiosity and information-seeking also emerged as important motivators in the period leading up to the decision to undergo sleeve gastrectomy. These psychosocial factors occurred within a broader context that gradually shaped participants’ consideration of sleeve gastrectomy as a potential choice. Consistent with previous studies, participants reported strong emotions such as embarrassment, disappointment, powerlessness, loneliness, isolation, and despair, which closely linked to weight gain and the social environment.^2,3^ Our findings highlight that obesity is not merely a physical health condition but is also deeply influenced by psychosocial and emotional factors.

One of the main themes in our study was how weight gain affects psychosocial health, especially self-perception and social experiences. When participants were unable to control their weight, they felt demotivated and blamed themselves, with perceived failures intensifying their frustration. Many participants also internalised negative social attitudes about obesity, which fostered ongoing self-criticism and a growing sense of disconnection from their bodies. These findings are consistent with the results of Fulton et al.^27^ and Westbury et al,^8^ who reported that weight stigma negatively affects psychosocial health. Detachment from self-image and avoidance of appearance-related situations were key indicators of low self-esteem, often accompanied by hopelessness and despair.^8,28^ These emotional responses were closely linked to stigma and internalised weight bias.^3^ In our sample, the accumulation of these emotional difficulties influenced how participants evaluated their capacity to cope without clinical intervention and shaped their view of surgery as a more sustainable option.

Social stigma emerged as a powerful influence on daily life and relationships. Anticipation or experience of judgment and exclusion led to social withdrawal and increased isolation. This finding aligns with research showing that internalised weight stigma and fear of social rejection are key factors in the psychosocial challenges of obesity.^2,3,27^ Our results also support the idea that the emotional effects of stigma may outweigh physical limitations.^3,29,30^ While stigma was not always a direct trigger for seeking surgery, its persistent emotional burden contributed to the perception that non-surgical attempts were insufficient, thereby influencing decision-making.

Family dynamics played a dual role in the pre-surgery experiences, consistent with earlier research.^2,3,7,31^ Critical or unsupportive advice led to frustration and increased feelings of inadequacy, making weight management more difficult and intensifying negative emotions such as guilt and low self-esteem.^3,27^ In contrast, supportive family relationships fostered resilience and a sense of belonging. However, when families avoided open communication or did not discuss weight-related issues, participants felt isolated and found it harder to seek help.^21^ Our findings overlap with research showing that social support can reduce psychological distress linked to obesity.^24,29^ However, well-intentioned interventions may sometimes reinforce negative self-perceptions through weight stigma.^8,27^ These complex interactions also shaped how participants appraised non-surgical strategies and, for some, reinforced the perception that surgery offered a more structured and socially acceptable path to change.

Emotional eating appeared as a common coping strategy in the pre-surgery period, often triggered by guilt, regret, or other negative emotions. A similar cycle of emotional distress and unhealthy eating behaviours has also been reported in earlier studies.^11,12,32^ Psychological distress and internalised stigma have been identified as strong predictors of these patterns and as major barriers to sustained weight loss.^33^ Participants often interpreted the persistence of emotional eating as evidence that their own strategies were no longer effective, strengthening their motivation to consider sleeve gastrectomy.

The decision-making process was shaped by fear and uncertainty about the unknown, but also by relief at the possibility of change and hope for better health and well-being.^16,17^ Despite concerns about surgical risks and post-surgical adjustment, many viewed surgery as a last opportunity to regain control. Curiosity and information-seeking served as important coping strategies, with participants researching the procedure and consulting others who had undergone surgery. Our findings showed the importance of comprehensive pre-operative counselling that addresses emotional and informational needs.^34^ Accurate information helped reduce uncertainty and situate surgery within the context of participants’ prior psychosocial experiences.

Our study has several limitations. Although some data included post-surgery experiences, our study deliberately focused on the pre-surgery period, where evidence remains limited, to maintain a clear and targeted scope. This focus can be considered a study limitation. The small sample size may not capture the full variety of experiences among individuals seeking sleeve gastrectomy in different cultural, regional, or socio-economic contexts. The themes identified were shaped by the stories of participants and our own interpretative perspective, which may limit the transferability of the findings to other groups. Because the data were collected retrospectively and the time elapsed since surgery varied from one to five years among participants, their memories of their emotional state before surgery may have been influenced by their experiences after surgery. This variation may have led to recall bias. Our sample consisted of female and highly educated participants, which may limit the generalisability of the findings across gender and educational backgrounds. The overrepresentation of certain subgroups should be considered when interpreting the results. Future research should include larger and more diverse samples and adopt longitudinal designs to better understand how emotional and psychosocial experiences change over time.

Our research highlighted the important role of self-perception and internalised stigma in shaping psychological and behavioural responses to weight gain and obesity. By focusing on the underexplored pre-surgery phase and retrospectively examining the emotional and social experiences of participants, we identified how psychosocial difficulties accumulate over time and influence readiness for surgery. These insights underscore the need for stigma-free, psychosocially informed obesity care that addresses emotional health prior to surgical intervention.

Using inductive thematic analysis allowed us to identify themes directly from participants’ own stories, ensuring that our findings were grounded in lived experiences rather than abstract theory. Interventions to improve psychosocial health and support lasting weight management should prioritise body image, reduce stigma, and foster supportive social environments. In practice, this may include pre-surgical counselling that explicitly addresses body image concerns, self-blame, and emotional coping strategies, alongside medical preparation for surgery. Beyond individual psychological support, such interventions could also involve families and communities, for example, through family-based training programmes that promote non-judgmental communication and greater awareness of weight stigma.

The use of digital tools such as online peer-support groups, moderated forums, or accessible psychoeducational resources may help reach individuals who experience social withdrawal or shame. These supports should be adapted to cultural contexts and individual needs. Furthermore, training not only healthcare providers but also other social groups about weight stigma can help create more understanding and supportive environments for people living with obesity. Future research should assess which psychosocial and educational interventions are most effective during the pre-surgery period.

## Conclusion

Our study highlights the major psychosocial challenges associated with weight gain and obesity in the pre-surgery period, and how these experiences shape individuals’ readiness and decision-making regarding sleeve gastrectomy. These difficulties often accumulate over time and influence how individuals evaluate their ability to manage weight without clinical support. Coping strategies, such as emotional eating, further reinforce emotional distress, creating a cycle that makes weight management more challenging and may increase openness to considering surgical options. By focusing on the decision-making context, our findings underscore the importance of holistic, stigma-free, and psychosocially supportive care prior to surgery, as well as throughout obesity treatment. Future research should further continue to investigate these psychosocial factors to inform more targeted interventions.

## Supplemental Material

Supplemental material - ‘I Will Never Succeed’: An Inductive Thematic Analysis of Psychosocial Challenges Experienced Before Sleeve Gastrectomy Among Patients in Turkey Supplemental material for ‘I Will Never Succeed’: An Inductive Thematic Analysis of Psychosocial Challenges Experienced Before Sleeve Gastrectomy Among Patients in Turkey by Sabahat Ölcer, Mehmet Tayanc in Journal of Patient Experience
