# Supplementary material for: ‘I Will Never Succeed’: An Inductive Thematic Analysis of Psychosocial Challenges Experienced Before Sleeve Gastrectomy Among Patients in Turkey
Source: J Patient Exp. 2026 Jul 3;13:23743735261458021. doi: 10.1177/23743735261458021 (PMC13332300; doi:10.1177/23743735261458021)
Supplement: Supplemental material - ‘I Will Never Succeed’: An Inductive Thematic Analysis of Psychosocial Challenges Experienced Before Sleeve Gastrectomy Among Patients in Turkey [file sj-pdf-1-jpx-10.1177_23743735261458021.pdf]

Supplementary file Table S1. Excerpts illustrating individuals' experiences, emotions and needs related to sleeve gastrectomy

Themes	Emotions	Quotations
**Self-perception and emotional responses to weight gain**	shame, frustration, helplessness, self-criticism, sadness, denial, alienation, despair	"Why did I do this to myself? I always blamed myself by saying, 'Why did not you stop the weight gain? If you had a problem, you should have handled it in another way.'" (SG1, female, 39, primary school, housewife)<br><br>"Because I gained and lost a lot of weight, my body shape became distorted. I did not want to see myself in the mirror. I avoided taking photos. I used to think I was very beautiful, but now when I look in the mirror, I cannot recognise myself." (SG2, female, 35, bachelor degree, civil servant)<br><br>"Up to a certain point, I did not really care about gaining weight, but one day I looked in the mirror and could not recognise myself. That day, I truly realised how much I had changed, and I felt very sad." (SG8, male, 24, bachelor degree, civil servant)
**Social experiences and stigma related to obesity**	embarrassment, social withdrawal, isolation, anger, resentment, humiliation	"Every time I stepped into a store, they would turn me away saying, 'We won't have clothes in your size.'" (SG1, female, 39, primary school, housewife)<br><br>"When people joked about my weight in social settings, I would smile, but deep down I would feel devastated. I obsessed over what others thought of me." (SG12, female, 31, vocational school degree, cosmetician)<br><br>"It made me angry when people looked at me with pity. There were times when I was the subject of jokes. Even being called 'fat' had started to feel unbearable." (SG19, male, 51, secondary school, retired)
**Family dynamics and support**	frustration, validation, loneliness, gratitude, frustration-anger mix	"My family would always say, 'Lose weight, be careful,' but they did it more by criticising, not in a motivating way." (SG13, female, 28, bachelor degree, teacher)<br><br>"My mom tried to be supportive, but some people around me criticised my weight gain, making me feel even worse." (SG16, male, 51, secondary school, photographer)<br><br>"My mom always said, 'You are beautiful as you are,' but I did not feel that way. Instead of offering support, they sometimes avoided talking about the issue altogether." (SG18, male, 56, primary school, driver)
**Emotional coping mechanisms and eating behaviours**	relief, helplessness, sadness, sense of escape	"When I was upset, I would always turn to food. Even if I was full, my hands kept reaching for snacks. I ate to suppress my emotions." (SG2, female, 35, bachelor degree, civil servant)<br><br>"Whenever I felt sad, I would catch myself eating. Fast food was a regular part of my life. It was the only thing that made me feel better." (SG13, female, 28, bachelor degree, teacher)<br><br>"Once I stopped smoking, I leaned on food like never before. When money and emotions weighed me down, eating became my only escape." (SG19, male, 51, secondary school, retired)
**Psychological barriers to**	hopelessness, exhaustion,	"I was dieting and losing weight, but I kept gaining it back. In the end, I said, 'I will never succeed.'" (SG8, male, 24,

**weight loss**	ambivalence, resignation	bachelor degree, civil servant)<br><br>“I have lost count of how many diets I have tried. Each time, I thought I would succeed but after a few months, everything went back to how it was. In the end, I gave up.” (SG12, female, 31, vocational school degree, cosmetician)<br><br>“I dieted for 3 months and lost 20 kilos, but then I gained it all back. The constant hunger from dieting exhausted me.” (SG14, female, 51, bachelor degree, housewife)<br><br>“I tried everything; online pills, workouts, professional diets. But I could not sustain any of it. Between life’s demands and work stress, everything fell through.” (SG19, male, 51, secondary school, retired)
**Decision-making process and information seeking about surgery**	fear, hope, ambivalence, curiosity, anxiety, relief, hesitation	“Online, the process was portrayed as simple. But after talking to people who had had surgery, I understood how challenging the process really was.” (SG7, male, 35, bachelor degree, self-employment)<br><br>“I always thought surgery was an unlikely option, but I wanted to lose weight and regain my mobility. When I heard an acquaintance had undergone surgery, I started considering it myself.” (SG13, female, 28, bachelor degree, teacher)<br><br>“I did not tell anyone about my decision. People might have scared me off, and I could have backed out. That is why I kept it secret.” (SG14, female, 51, bachelor degree, housewife)<br><br>“At first, the idea of surgery seemed terrifying to me. But after talking to people who had undergone it, I realised the process is tough but the results are worth it.” (SG16, male, 51, secondary school, photographer)<br><br>“My brother had undergone surgery, and seeing him made me consider it too. His success story motivated me. I also decided to do it for a happier life with my spouse.” (SG19, male, 51, secondary school, retired)
**Impact of weight on psychosocial health**	sadness, loneliness, frustration, despair, inadequacy	“I no longer wanted to spend time with people. Cancelling plans became routine because I felt terrible about myself.” (SG3, female, 28, high school, civil servant)<br><br>“I used to love going out, but as I gained weight, I started preferring to stay home. I isolated myself from people.” (SG15, male, 46, master degree, civil servant)<br><br>“I used to be more confident. I would go play soccer with my friends. But as I gained weight, I stopped even wanting to go out. At work, I avoided drawing attention to myself and never let anyone discuss my weight.” (SG19, male, 51, secondary school, retired)
